# Using genome wide association studies to identify common QTL regions in three different genetic backgrounds based on Iberian pig breed

**DOI:** 10.1371/journal.pone.0190184

**Published:** 2018-03-09

**Authors:** Ángel M. Martínez-Montes, Almudena Fernández, María Muñoz, Jose Luis Noguera, Josep M. Folch, Ana I. Fernández

**Affiliations:** 1 Departamento de Genética Animal, Instituto Nacional de Investigación y Tecnología Agraria y Alimentaria (INIA), Madrid, Spain; 2 Centro de I+D en Cerdo Ibérico, Zafra, Badajoz, Spain; 3 Departament de Genètica i Millora Animal, Institut de Recerca i Tecnologia Agroalimentàries (IRTA), Lleida, Spain; 4 Departament de Ciència Animal i dels Aliments, Facultat de Veterinària, Universitat Autònoma de Barcelona (UAB), Bellaterra, Spain; 5 Plant and Animal Genomics, Centre de Recerca en Agrigenòmica (CRAG), Consorci CSIC-IRTA-UAB-UB, Campus UAB, Bellaterra, Spain; Universita degli Studi di Bologna, ITALY

## Abstract

One of the major limitation for the application of QTL results in pig breeding and QTN identification has been the limited number of QTL effects validated in different animal material. The aim of the current work was to validate QTL regions through joint and specific genome wide association and haplotype analyses for growth, fatness and premier cut weights in three different genetic backgrounds, backcrosses based on Iberian pigs, which has a major role in the analysis due to its high productive relevance. The results revealed nine common QTL regions, three segregating in all three backcrosses on SSC1, 0–3 Mb, for body weight, on SSC2, 3–9 Mb, for loin bone-in weight, and on SSC7, 3 Mb, for shoulder weight, and six segregating in two of the three backcrosses, on SSC2, SSC4, SSC6 and SSC10 for backfat thickness, shoulder and ham weights. Besides, 18 QTL regions were specifically identified in one of the three backcrosses, five identified only in BC_LD, seven in BC_DU and six in BC_PI. Beyond identifying and validating QTL, candidate genes and gene variants within the most interesting regions have been explored using functional annotation, gene expression data and SNP identification from RNA-Seq data. The results allowed us to propose a promising list of candidate mutations, those identified in *PDE10A*, *DHCR7*, *MFN2 and CCNY* genes located within the common QTL regions and those identified near *ssc-mir-103-1* considered *PANK3* regulators to be further analysed.

## Introduction

QTL identification is one of the most relevant approaches used in livestock genomic studies in order to understand the genetic architecture that regulates complex productive traits. To date, different porcine breed schemes had been used for QTL scanning, from simple designs including purebred populations such as Pietrain, Landrace or Duroc [1,2], to more complex schemes, mating different breeds in order to compare animals with diverse phenotypes, as Duroc x Pietrain [3,4], Iberian x Landrace [5] or three-way crosses such as Duroc x (Landrace x Large White) [6]. These studies have reported a large number of QTL for different productive traits such as growth (1,328), fat composition (1,311), drip loss (1,071), average daily gain (568), average backfat thickness (332) or intramuscular fat content (244) (PigQTLdb) [7].

In spite of the great amount of QTL identified in multiple pig breeds, the application of the results in pig breeding and the identification of causal genes and mutations (QTN) has not been very successful. One of the major limitations had been the low number of available markers [8,9]. However, this issue has been settled with the development of high-density genotyping platforms, which provide a high number of markers along the genome, allowing us to QTL fine-map and to conduct genome-wide analysis (GWAS) [10,11]. Another major limitation for QTN identification has been the limited number of animals employed in the analyses [12–14], reporting unreliable results that cannot be validated in different genetic backgrounds. So far, few porcine QTL regions related to productive traits have been confirmed in different animal material, some exceptions are the QTL around *LEPR* region for growth, fatness and meat quality traits identified in Iberian x Landrace cross [15], and validated in Iberian x Meishan cross [16] and Duroc populations [17,18], the FAT1 QTL located on SSC4 associated with fatty acid metabolism validated in Meishan x Large White, Iberian x Landrace and Wild Boar intercrosses [19–23], the QTL around *MC4R* for performance traits [24] and the QTL located on SSC12 for fatty acid composition was validated in different Iberian and Landrace cross populations and in purebred Duroc [17,25–28].

The aim of the current work was to validate QTL regions through GWAS analyses for growth, fatness and premier cut yields in three different genetic backgrounds F1 (Iberian x Landrace) x Landrace (BC_LD), F1 (Iberian x Duroc) x Duroc (BC_DU) and F1 (Iberian x Pietrain) x Pietrain (BC_PI) backcrosses. Here, Iberian background had a major role in the analysis due to the high productive relevance of this breed [29]. Beyond identifying and validating QTL, candidate genes and polymorphism within the most interesting regions have been proposed and explored.

## Material and methods

### Animals

**Fig 1:**
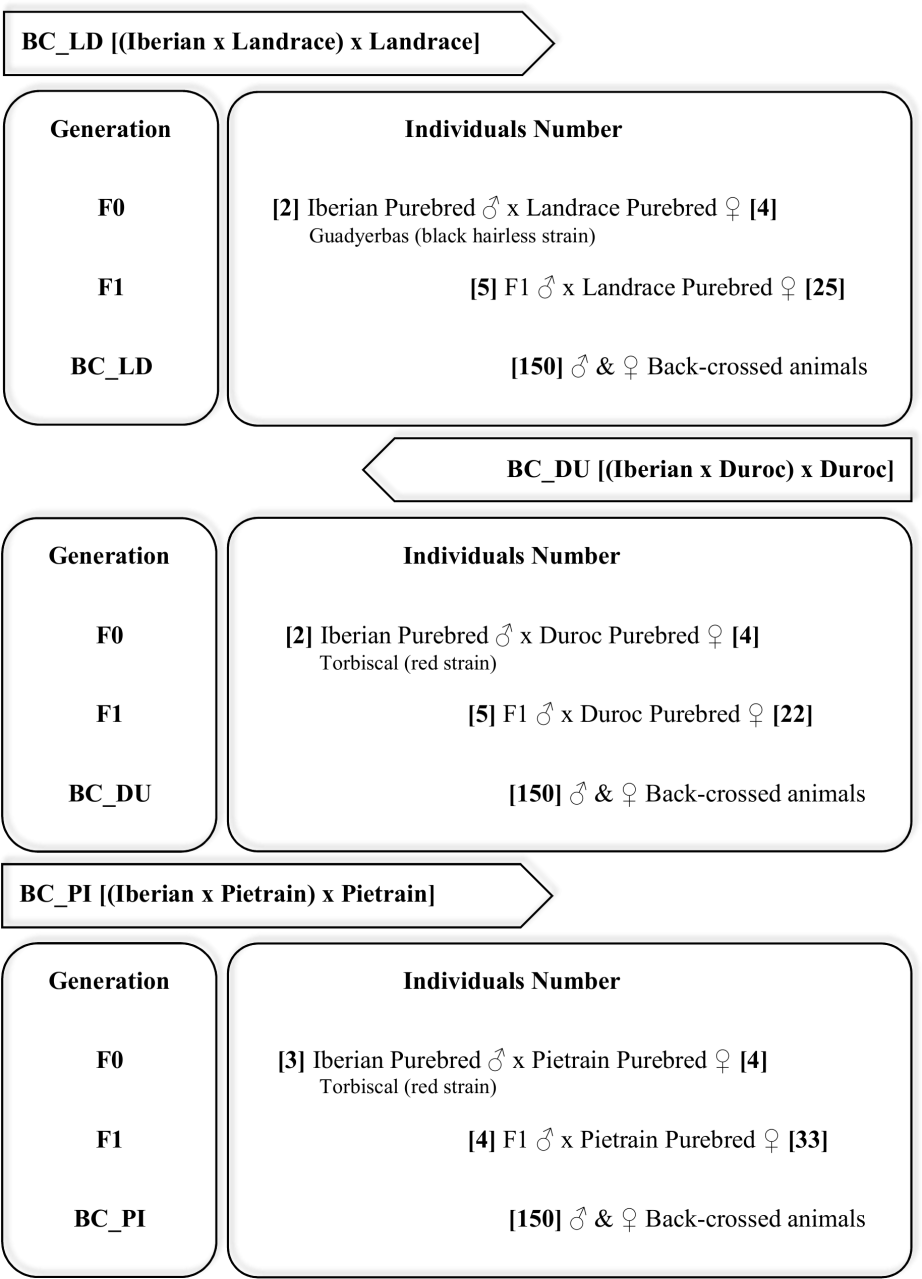
Backcross generation scheme: Schematic representation of each of the backcrosses used (BC_LD, BC_DU and BC_PI), specifying the number of individuals in each generation.

Phenotypic and genotypic data used in this study belong to three different experimental backcrosses: F1 (Iberian x Landrace) x Landrace, F1 (Iberian x Duroc) x Duroc and F1 (Iberian x Pietrain) x Pietrain. The Iberian parental belong to different Iberian strains, Guadyerbas (black hairless strain), used for the Iberian x Landrace backcross, and Torbiscal (red strain), used for the Iberian x Duroc and Iberian x Pietrain backcrosses, which differ, apart from the coat colour, in productive traits such as growth ratio, backfat thickness and premium cut yields [30]. Schematic representation of the backcross generation is shown in Fig 1. All pigs were raised and fed under the standard, intensive system in Europe; males were not castrated. After a suckling period of between 23 and 28 d, piglets were allocated in pens with 12 individuals in each pen and were given ad libitum access to a pelleted diet (13.4 MJ/kg of ME, 18.3% of CP, 1.2% of lysine). When the piglets were about 75 d old, they were moved to a fattening building. They were penned in groups of 10 to 12 animals separated by sex, and during the whole test period they had ad libitum access to a cereal based commercial diet (13.4 MJ/kg of ME, 17.5% CP, 1% lysine). Pigs tested at the same time and in the same fattening building were considered as 1 contemporary group (batch).

#### Ethics statement

All animal procedures were performed according to the Spanish Policy for Animal Protection RD1201/05, which meets the European Union Directive 86/609 about the protection of animals used in experimentation. The protocol was approved by the Committee on the Ethics of Animal Experiments of the Instituto Nacional de Investigación y Tecnología Agraria y Alimentaria CEEA (Permit Number: 2014/026).

### Phenotypic data

Seven traits related to growth, fatness and premium cut yields recorded for all three backcross pigs were analyzed (Table 1). These traits were: body weight at 150 days of mean age (BW150), backfat thickness measured at 75 kg of live weight (BFT75) and at slaughter (BFTS), mean weights (left and right) of premium cuts, hams (HW) shoulders (SW) and loin bone-in (LBW), and intramuscular fat content (IMF) measured in *Longissimus dorsi* samples at slaughter as described in Fernández et al. [31].

**Table 1:** Phenotypic traits recorded for the backcrossed pigs analyzed. Number of individuals (N), mean and standard deviation (SD) were calculated. Measures of body weight at 150 days of mean age (BW150), backfat thickness measured at 75 kg (BFT75) and at slaughter (BFTS), mean weights of hams (HW) shoulders (SW) and loin bone-in (LBW) and intramuscular fat content (IMF) in *Longissimus dorsi* muscle.

	BW150	BFT75	BFTS	HW	SW	LBW	IMF
Merged							
N	384	270	280	381	381	372	369
Mean	70.25	12.55	1.95	10.01	5.38	6.51	2.65
SD	12.09	2.511	0.96	1.32	0.75	1.01	1.34
BC_LD							
N	101	101	79	99	99	98	86
Mean	77.27	12.59	2.44	9.99	5.31	6.91	2.02
SD	10.07	1.47	0.66	1.35	0.79	0.99	0.63
BC_DU							
N	135	55	139	135	135	129	135
Mean	73.57	16.08	2.49	9.9	5.51	6.12	3.86
SD	10.11	1.72	0.95	1.33	0.73	0.87	1.32
BC_PI							
N	142	112	141	141	141	139	142
Mean	62.18	10.79	1.42	10.14	5.3	6.59	1.9
SD	10.62	1.64	0.6	1.29	0.73	1.03	0.75

### Genotypic data

Two different genotyping platforms were used, BC_LD and BC_PI backcrosses were genotyped with the platform PorcineSNP60 BeadChip (Illumina, Inc.) [32], containing 64,232 SNPs. GenomeStudio software (Illumina, Inc.) was employed to visualize, edit, standard quality filter and extract genotyping data. Backcross BC_DU was genotyped with Axiom® Porcine Genotyping Array (Affymetrix, Inc.) [33], containing 658,692 SNPs. Axiom™Analysis Suite 2.0 was employed to visualize, quality filter and extract genotype data. For individual analysis of each backcross, additional data filtering was carried out using GenABEL package [34] in R environment, and polymorphisms with a minimum allele frequency (MAF) < 0.05 were discarded for further analysis. In order to be able to compare data from the two platforms SNPchiMp v.3 [35] was used to select SNPs overlapping between both.

### GWAS analysis

Genome-wide association analysis was conducted in a joint analysis of the three backcrosses, merged dataset. Moreover, in order to identify the association source, specific genome-wide association analyses were conducted for each backcross. The analyses were performed with GenABEL R package using the following the model:

$$y_{ijk}=S_{i}+B_{j}+bx_{k}+\sum_{l}\lambda_{lk}a_{l}+u_{k}+e_{ijk}$$

where *y*_*ijk*_ is the trait value of *k*th individual, *S*_*i*_ and *B*_*j*_ are fixed effects for sex and batch respectively and *b* is the carcass weight regression coefficient only included for BFTS, HW, SW, LBW and IMF traits. Additive effect of the SNP is *a*_*l*_ and *λ*_*lk*_ is the indicator related with the number of copies of the *l*th allele (0, 1 or 2), *u*_*k*_ is the infinitesimal genetic random effect of the *k*th individual, with covariance Aσ_u_^2^, A being the numerator relationship matrix and *e*_*ijk*_ is the random residual term. For the joint analysis (merged database) the backcross was included as fixed effect. A q-value <0.05 [36] was employed to identify significant associations.

### Region analysis

QTL regions were determined by two or more consecutive SNPs significantly associated with each phenotypic trait, to a maximum distance of 15Mb. Associated regions identified in the merged database were considered *a priori* common QTL.

### Haplotype analysis

Those significant SNPs within QTL regions identified in the merged dataset analysis were used for haplotype association. Haplotypes were built using Phase v2.1 [37] and association analyses were conducted following the previously described model with Qxpak 5.0 software [38].

### Candidate genes

Gene content of the QTL regions were determined using Biomart tool from the Porcine Ensembl database, *Sscrofa11*.*1*. In order to identify potential candidate genes the function of genes within each of the selected regions (±1Mb) were examined using VarElect [39], STRING [40] Babelomics [41] and ncbi databases (growth, muscle development and fat metabolism related genes). Moreover, additional information of gene expression data from a previous study in BC_LD [42] and a parallel RNA-Seq study conducted in the BC_LD and BC_PI pigs with divergent phenotypes for the same analyzed traits [43] was also used. Genes mapped within the selected QTL regions showing significant differential expression (q-value< 0.05 and fold change> 1.5) were retained.

### SNP calling

RNA-Seq data obtained from liver, hypothalamus and *Longissimus dorsi* muscle samples, from pigs with divergent phenotypes for growth and fatness on each backcross [43,44] was used for SNP calling, in order to identify candidate polymorphism within the selected candidate genes. RNA-Seq data filtering, mapping and SNP calling was carried out with CLC Genomics Workbench (www.clcbio.com). Reads were mapped against the pig reference genome *Sscrofa10*.*2*, and re-annotated for *Sscrofa 11*.*1*. Mapping parameters were set at a cost of two for mismatches per read, cost of three for insertion or deletions, a length fraction of 0.9 and a similarity fraction of 0.8. Quality-based variant detection tool from CLC Genomic Workbench, based on neighborhood quality [45,46], was used to perform SNP calling, setting a neighborhood radius of 5, minimum neighborhood quality of 15 and minimum central quality of 20, a minimum coverage of 3 and a 20% of minimum variant frequency.

The SNPs were classified using VENNY 2.1 tool [47] as: 1) SNPs identified in genes located in common regions observed in all the backcrosses, and 2) SNPs segregating in the backcross where backcross-specific QTL regions were detected. Besides, the functional relevance of these SNPs was analyzed with VeP tool (Variant effect Prediction, Ensembl) in order to identify potential candidate mutations.

## Results

### SNP data

For the GWAS analyses only SNPs overlapping between genotyping platforms (60K and 650K) for each backcross were retained. A total of 39,279 SNPs in 102 BC_LD pigs, 38,684 SNPs in 139 BC_DU pigs, and 38,891 SNPs in 144 individuals BC_PI pigs were selected. The merged dataset contained 40,929 SNPs in 385 backcrossed pigs. The SNPs showing a MAF ≥ 0.05 were around 80% in each backcross and 97% in the merged dataset. Approximately a 50% of them showed intermediate frequency (MAF ≥ 0.25) in each backcross, and 61% in the merged dataset (S1 Fig). These data revealed a similar allele frequencies distribution across all the animal material, therefore none restraining effects related to SNP informativity would be expected in the QTL region identification, although it could be found for specific genomic regions.

### GWAS analysis

The GWAS analyses for BW150, BFT75, BFTS, IMF, HW, SW and LBW traits were carried out for the merged dataset and independently for each backcross. Significance associations were considered those with q-value lower to 0.05 (S2 Table). The results from the merged analysis reported a total of 89 significant associations (TAS), 22 TAS in the specific BC_LD analysis, 21 TAS in the specific BC_DU analysis and 32 TAS in the specific BC_PI (S1 Table).

### Region analysis

Region determination following previously defined criteria revealed 15 QTL regions in the merged dataset, *a priori* considered common QTL regions, eight of which were not identified in any specific backcross analyses (Table 2). Additionally, another four QTL regions were specifically identified in the BC_LD, another four regions in the BC_DU and four regions in the BC_PI (Table 3).

**Table 2:** Significant QTL regions identified in the merged dataset or in the merged dataset and individual backcross GWAS. Results for body weight at 150 days (BW150), backfat thickness at slaughter (BFTS), ham (HW), shoulder (SH), loin bone-in (LBW) mean weights and intramuscular fat (IMF): region name, dataset, genomic position, number of associated SNPs, associated trait, additive effects and *P*-value. The effect was estimated on the most significant SNP of each QTL region.

Region	Dataset *	Genomic Position	SNPs	Trait	a ± se	*P-value*
M1	m	1:373,626–381,075	2	LBW	0.20 ± 0.06	*4*.*2 ×10*^*-5*^
M2	m+ld	1:768,502–2,754,517	2	BW150	−1.68 ± 0.49	*1*.*1 ×10*^*-4*^
M3	m	1:43,378,688–52,039,046	2	BFTS	−0.35 ± 0.08	*4*.*4 ×10*^*-6*^
M4	m+pi	2:145,257–8,936,150	4	BFTS	0.32 ± 0.08	*1*.*9 ×10*^*-5*^
M5	m	2:3,321,699–9,006,820	2	LBW	0.21 ± 0.06	*8*.*9 ×10*^*-5*^
M6	m	4:8,968,669–18,736,459	3	SW	0.09 ± 0.03	*1*.*2 ×10*^*-4*^
M7	m	4:106,675,400–114,087,749	3	BFTS	0.23 ± 0.06	*3*.*2 ×10*^*-5*^
M8	m	5:45,418,338–58,984,249	2	BFTS	0.57 ± 0.15	*8*.*8 ×10*^*-5*^
M9	m+pi	6:20,539224–39,457626	7	HW	0.14 ± 0.03	*1*.*3 ×10*^*-5*^
M10	m+du	6:25,753,694–30,429,968	4	LBW	−0.12 ± 0.03	*2*.*5 ×10*^*-5*^
M11	m+ld	6:71,901,978–86,757,750	2	SW	−0.09 ± 0.02	*2*.*7 ×10*^*-5*^
M12	m+ld	6:80,622,284–112,485,359	6	HW	−0.13 ± 0.03	*3*.*8 ×10*^*-5*^
M13	m	7:3,508,262–3,831,582	2	SW	0.08 ± 0.02	*3*.*2 ×10*^*-5*^
M14	m	10:57,593,745–57,794,053	3	HW	−0.17 ± 0.04	*1*.*6 ×10*^*-5*^
M15	m+du	13:14,170,761–24,633,705	8	IMF	−0.28 ± 0.07	*1*.*4 ×10*^*-5*^

*: m: merged; ld: BC_LD; du: BC_DU; pi: BC_PI.

**Table 3:** Backcross specific QTL regions. Results for body weight at 150 days (BW150), backfat thickness at 75 kg (BFT75), backfat thickness at slaughter (BFTS), ham weight (HW), shoulder weight (SH), loin bone-in weight (LBW) and intramuscular fat (IMF): region name, backcross, genomic position, number of associated SNPs, associated trait, additive effects and *P*-value. The effect was estimated on the most significant marker of each QTL region.

Region	BC	Genomic Position	SNPs	Trait	a ± se	*P-value*
LD1	BC_LD	5:5,196,795–9,634,200	6	LBW	0.13 ± 0.04	*4*.*6 ×10*^*-5*^
LD2	BC_LD	6:33,958,464–33,971,693	2	SW	-0.10 ± 0.02	*2*.*2 ×10*^*-5*^
LD3	BC_LD	11:27,479,379–28,373,530	2	BFT75	0.82 ± 0.20	*1*.*2 ×10*^*-4*^
LD4	BC_LD	16:53,929,823–59,747,430	3	BFTS	-0.47 ± 0.12	*7*.*7 ×10*^*-5*^
DU1	BC_DU	4:129,180,275–129,209,724	2	BFT75	-1.72 ± 0.50	*1*.*8 ×10*^*-4*^
DU2	BC_DU	8:20,674,494–20,770,188	2	BW150	-2.81 ± 0.73	*1*.*6 ×10*^*-4*^
DU3	BC_DU	9:9,566,683–9,898,353	2	BFTS	0.45 ± 0.11	*2*.*4 ×10*^*-5*^
DU4	BC_DU	15:8,659,709–9,791,368	10	SW	0.15 ± 0.04	*1*.*8 ×10*^*-4*^
PI1	BC_PI	2:44,838,080–47,326,959	7	BW150	-3.84 ± 0.98	*1*.*1 ×10*^*-4*^
PI2	BC_PI	4: 73,710,930–79,915,989	7	BFTS	0.35 ± 0.08	*2*.*6 ×10*^*-5*^
PI3	BC_PI	11:14,6,72,532–14,737,228	3	SW	0.27 ± 0.06	*7*.*9 ×10*^*-6*^
PI4	BC_PI	13:190,486,890–191,164,340	2	IMF	0.32 ± 0.09	*4*.*1 ×10*^*-5*^

The QTL regions identified in the merged dataset, located on SSC1, SSC2, SSC4, SSC5, SSC6, SSC7, SSC10 and SSC13, were associated with all phenotypic traits except for BFT75 (Table 2). The eight QTL regions identified in the merged dataset but not in the individual analyses were localized on SSC1, SSC2, SSC4, SSC5, SSC7 and SSC10, associated with BFTS, HW, SW and LBW (Table 2). The four regions identified specifically in the BC_LD were located on SSC5, SSC6, SSC11 and SSC16 associated with BFT, SW and LBW (Table 3). Four regions identified specifically in the BC_DU were located on SSC4, SSC8, SSC9, and SSC15, and showed association with BW150, BFT and SW (Table 3). Finally, the four regions identified specifically in the BC_PI were located on SSC2, SSC4, SSC11 and SSC13, and showed association with BW150, BFTS, IMF and SW (Table 3).

### Haplotype analyses

For the QTL regions identified in the merged dataset, haplotypes were constructed and analyzed taking into account all the SNPs significantly associated in order to validate the QTL origin. The haplotype description and segregation is shown in S2 Table. Results of the haplotype association analyses in the merged dataset and each backcross are summarized in Table 4.

**Table 4:** Results of the haplotype association analysis for the common QTL regions in the merged dataset and each individual backcross.

Region	Trait	SNP	Haplotypes>1%	Association test	
				Dataset	*P*-value
M1 (1:0.3–0.4)	LBW	2	3	Merged	4.3 ×10^-4^
				BC_LD	0.518
				BC_DU	NA
				BC_PI	1.3 ×10^-3^
M2 (1:0–3)	BW150	2	3	Merged	1.7 ×10^-6^
				BC_LD	2.5 ×10^-3^
				BC_DU	0.034
				BC_PI	1.1 ×10^-4^
M3 (1:43–52)	BFTS	2	3	Merged	3.3 ×10^-4^
				BC_LD	NA
				BC_DU	NA
				BC_PI	2.4 ×10^-4^
M4 (2:0–9)	BFTS	4	7	Merged	1.9 ×10^-6^
				BC_LD	NA
				BC_DU	2.8 ×10^-3^
				BC_PI	2.5 ×10^-4^
M5 (2:3–9)	LBW	2	4	Merged	1.7 ×10^-7^
				BC_LD	4.4 ×10^-4^
				BC_DU	4.4 ×10^-3^
				BC_PI	0.015
M6 (4:9–19)	SW	3	8	Merged	5.0 ×10^-6^
				BC_LD	0.010
				BC_DU	0.018
				BC_PI	0.266
M7 (4:107–114)	BFTS	3	5	Merged	1.9 ×10^-5^
				BC_LD	NA
				BC_DU	6.2 ×10^-3^
				BC_PI	5.7 ×10^-3^
M8 (5:45–59)	BFTS	2	3	Merged	9.0 ×10^-6^
				BC_LD	NA
				BC_DU	1.2 ×10^-3^
				BC_PI	0.376
M9 (6:21–39)	HW	7	8	Merged	1.7 ×10^-5^
				BC_LD	0.086
				BC_DU	0.044
				BC_PI	0.028
M10 (6:26–30)	LBW	4	8	Merged	8.8 ×10^-4^
				BC_LD	0.296
				BC_DU	6.7 ×10^-3^
				BC_PI	0.496
M11 (6:72–87)	SW	2	4	Merged	5.5 ×10^-6^
				BC_LD	0.031
				BC_DU	0.017
				BC_PI	0.157
M12 (6:81–112)	HW	6	13	Merged	2.8 ×10^-8^
				BC_LD	1.1 ×10^-4^
				BC_DU	0.161
				BC_PI	0.738
M13 (7:3–4)	SW	2	4	Merged	1.5 ×10^-5^
				BC_LD	0.031
				BC_DU	9.9 ×10^-4^
				BC_PI	5.8 ×10^-3^
M14 (10:57–58)	HW	3	3	Merged	2.9 ×10^-4^
				BC_LD	9.0 ×10^-3^
				BC_DU	0.365
				BC_PI	0.028
M15 (13:14–25)	IMF	8	21	Merged	4.3 ×10^-4^
				BC_LD	0.257
				BC_DU	2.6 ×10^-4^
				BC_PI	0.067

NA: missing genotypes or one single haplotype segregating in the backcross.

The M2, M5 and M13 QTL regions appeared segregating in all three backcrosses, M6, and M11 in BC_LD and BC_DU, the M4, M7 and M9 in BC_PI and BC_DU, and the M14 in BC_LD and BC_PI, and all those were considered actual common QTL regions (Table 5). Nonetheless, M12 appeared only segregating in BC_LD, M8 and M10 and M15 appeared only segregating in BC_DU, and M1 and M3 appeared only segregating in BC_PI, therefore these (M1, M3, M8, M10, M12 and M15) were considered backcross-specific QTL regions for further analysis.

**Table 5:** Common QTL regions identified. QTL detected for body weight at 150 days (BW150), ham weight (HW), shoulder weight (SH), loin bone-in weight (LBW) and intramuscular fat (IMF): region name, associated trait, genomic position, dataset where the QTL was identified and actual backcross segregation.

Region	Trait	Genomic Position	DataSet	Actual segregation
M2	BW150	1:768,502–2,754,517	m+ld	BC_LD, BC_DU, BC_PI
M4	BFTS	2:145,257–8,936,150	m+pi	BC_DU, BC_PI
M5	LBW	2:3,321,699–9,006,820	m	BC_LD, BC_DU, BC_PI
M6	SW	4:8,968,669–18,736,459	m	BC_LD, BC_DU
M7	BFTS	4:106,675,400–114,087,749	m	BC_DU, BC_PI
M9	HW	6:20,539224–39,457626	m+pi	BC_DU, BC_PI
M11	SW	6:71,901,978–86,757,750	m+ld	BC_LD, BC_DU
M13	SW	7:3,508,262–3,831,582	m	BC_LD, BC_DU, BC_PI
M14	HW	10:57,593,745–57,794,053	m	BC_LD, BC_PI

### Candidate genes and SNPs

To identify positional candidate genes, genes located within significant QTL regions were annotated using BioMart tool. In the common QTL regions 729 genes were identified, and 180 genes on the backcross-specific regions. To focus in a more affordable candidate gene list, criteria based on functionality, examined with Fatigo and VarElect tools, and gene expression differences from parallel RNA-Seq studies (divergent pigs for the analyzed traits) [42,43] were used to prioritize them. A total of 85 candidate genes were selected, 50 genes for the common regions and 35 genes for specific backcross regions (Table 6).

**Table 6:** Functional candidate genes identified in the common and specific QTL regions.

Region	Gene
Common QTL region	
M2 (1:0.7–3)	*PDE10A*
M4 (2:0.1–9)	*HRAS*, *TNNI2*, *TNNT3*, *KCNQ1*, *DHCR7*, *FGF3*, *FGF4*, *FGF19*, *CPT1A*, *GAL*, *ACTN3*, *LTBP3*, *MEN1*, *VEGFB*, *SLC22A8 DE*
M5 (2:3–9)	*FGF3*, *FGF4*, *FGF19*, *DHCR7*, *CPT1A*, *GAL*, *ACTN3*, *LTBP3*, *MEN1*, *VEGFB*, *SLC22A8 DE*
M6 (4:8–19)	*MYC*, *FBXO32*, *COL14A1 DE*
M7 (4:106–114)	*RAP1A*, *CSF1*, *PPM1J DE*
M9 (6:20–40)	*FTO*, *NOD2*, *SLC9A5 DE*
M11 (6:71–88)	*NPPA*, *NPPB*, *MFN2*, *CASP9*, *PAX7*, *HSPG2*, *CDC42*, *HMGCL*, *TRIM63*, *SLC9A1*, *FABP3*
M13 (7:3.5–4)	*PPP1R3G*, *BMP6*, *DSP DE*
M14 (10:57–58)	*CCNY*
QTL backcross-specific	
BC_LD specific	
LD1(5:5–10)	*A4GALT*, *ACO2*, *ATF4*, *CYB5R3*, *EP300*, *MCAT*, *MKL1*, *NAGA*, *PDGFB*, *SREBF2*, *TSPO*
LD2(6:32–34)	*SALL1*
LD4 (16:53–59)	*PANK3*
M12 (6:80–113)	*FNDC5*, *MPPE1DE*
BC_DU specific	
DU1 (4:128–130)	*LMO4*
DU2 (8:20–21)	*RBPJ*
DU3 (9: 8–10)	*NEU3*, *UPC3*
M8 (5:45–59)	*GLY2*
M10 (6:25–33)	*AGRP*, *FTO*, *KCTD19*, *LCAT*, *MMP2*, *SLC9A5 DE*
M15 (13:14–24)	*GPD1L*, *DCLK3 DE*, *ARPP21 DE*
BC_PI specific	
PI1 (2:44–48)	*CYP2R1*, *PDE3B*, *COPB1*
PI2 (4:73–80)	*CYP7A1*
M1 (1:0–0.5)	*DLL1*
M3 (1:43–52)	*PLN*

^DE^ Genes differentially expressed (Fold change >1.5) in RNA-Seq analysis [42,43]; M: common QTL regions coming from the merged dataset; LD: specific QTL region coming from the BC_LD analysis; DU: specific QTL region coming from the BC_DU; and PI: specific QTL region coming from the BC_PI analysis.

Moreover, candidate SNPs were selected from those SNPs identified in parallel RNA-Seq studies [42,43] within candidate genes and segregating, at least 10% of the pigs carrying the alternative allele in the corresponding backcrosses (S3 Table). A total of 46 out from 62 segregating SNPs filtered with VeP tool were considered potentially relevant (Table 7).

**Table 7:** Most relevant SNPs considered within selected candidate genes. dbSNP identification, gene name, predicted effect, QTL region.

SNP ID	Gene	Effect Prediction	QTL region
rs321518792	*PDE10A*	—	M2
rs334446395	*DHCR7*	missense_variant	M4
rs323256060	*DHCR7*	missense_variant	M4
rs331309328	*VEGFB*	3_prime_UTR_variant	M4, M5
rs319115249	*VEGFB*	3_prime_UTR_variant	M4, M5
rs330540333	*VEGFB*	3_prime_UTR_variant	M4, M5
rs320791982	*MEN1*	3_prime_UTR_variant	M5
rs338285951	*MEN1*	3_prime_UTR_variant	M5
rs340867100	*MEN1*	3_prime_UTR_variant	M5
rs321008722	*COL14A1*	3_prime_UTR_variant	M6
rs342512843	*CSF1*	missense_variant	M7
rs318724354	*CSF1*	5_prime_UTR_variant	M7
rs334648266	*CASP9*	3_prime_UTR_variant	M11
rs343272675	*CDC42*	3_prime_UTR_variant	M11
rs341725204	*MFN2*	3_prime_UTR_variant	M11
rs320903047	*MFN2*	3_prime_UTR_variant	M11
rs341521028	*MFN2*	3_prime_UTR_variant	M11
rs323861488	*MFN2*	3_prime_UTR_variant	M11
rs335545199	*MFN2*	3_prime_UTR_variant	M11
rs329360100	*BMP6*	3_prime_UTR_variant	M13
rs333159364	*BMP6*	3_prime_UTR_variant	M13
rs328999141	*BMP6*	3_prime_UTR_variant	M13
rs333365084	*BMP6*	3_prime_UTR_variant	M13
rs324511668	*BMP6*	3_prime_UTR_variant	M13
rs342273848	*BMP6*	3_prime_UTR_variant	M13
rs325292425	*BMP6*	3_prime_UTR_variant	M13
rs337979499	*BMP6*	3_prime_UTR_variant	M13
rs337148204	*BMP6*	3_prime_UTR_variant	M13
rs321518849	*BMP6*	3_prime_UTR_variant	M13
rs336783880	*BMP6*	3_prime_UTR_variant	M13
rs320422417	*BMP6*	downstream_gene_variant	M13
rs339868002	*DSP*	3_prime_UTR_variant	M13
rs322085085	*DSP*	3_prime_UTR_variant	M13
*7:4913389A>C	*DSP*	missense_variant	M13
rs332415316	*CCNY*	3_prime_UTR_variant	M14
rs332415316	*CCNY*	intron_variant	M14
rs690336573	*CCNY*	3_prime_UTR_variant	M14
rs330813245	*CCNY*	3_prime_UTR_variant	M14
rs332978767	*PANK3/mir-103-1*	downstream_gene_variant	LD4
rs341887311	*PANK3/mir-103-1*	downstream_gene_variant	LD4
rs334607455	*PANK3/mir-103-1*	downstream_gene_variant	LD4
rs324153600	*PANK3/mir-103-1*	downstream_gene_variant	LD4
rs335346325	*PANK3/mir-103-1*	downstream_gene_variant	LD4
rs320940204	*PANK3/mir-103-1*	downstream_gene_variant	LD4
rs337092527	*PANK3/mir-103-1*	downstream_gene_variant	LD4
rs698471988	*NEU3*	3_prime_UTR_variant	DU3

*identified in the current study, EVA accession number: PRJEB23068 (www.dev.ebi.ac.uk/eva/?eva-study=PRJEB23068)

## Discussion

The main objective of the current study was to validate QTL among different genetic backgrounds. In order to achieve this objective, a GWAS study was designed specifically for this purpose, in which three experimental backcrosses involving four different pig breeds were employed in a merged dataset and backcross-specific datasets. Here, it is worth to highlighting the productive value of the Iberian breed versus the widely employed commercial pig breeds Landrace, Duroc and Pietrain. The relevance of Iberian pig in meat production relies on the ability to produce high quality dry-cured products [48], due to particular characteristics associated with fattening, meat and growth processes [29]. Iberian pigs have high fat deposition and desaturation levels, with particular fatty acid profiles and tend to accumulate infiltrated fat in muscle mass [49]. Leptin resistance is a characteristic property identified in this breed, and is associated with high food intake [15,16].

Although linkage QTL mapping has been proved, and remains, a powerful method to identify regions of the genome that co-segregate with a given trait in experimental populations, limitations such as the assumption of alternative QTL alleles in the parental generation and the amount of recombination events need to fine map QTL are well known. GWAS overcome these two main limitations, while introducing other drawbacks, but it can provide insights into the genetic architecture of the trait and suggest candidate genes to be further analysed [50,51]. Even more, in the current study, the lack of SNP genotypes for some parents, the different genetic Iberian backgrounds together with the limited amount of expected recombinants due the population design were considered crucial criteria for choosing the GWAS approach.

The approach used in the current study enabled us to identify several associations between SNPs and productive traits, setting a great base for further analyses. Besides identifying TAS, we were able to define a total of 27 QTL regions in all the material. Here, the impact of the sample size in QTL scans can be shown, even merging different genetic backgrounds. The merged GWAS analysis allowed us to identify and validate common QTL, some of which were undetected in the specific backcross analysis, but also allowed us to identify some of the backcross-specific QTL regions such as the M1 and M3 in the BC_PI, M8, M10 and M15 in BC_DU and M12 in BC_LD. More importantly, nine common QTL were identified and validated through region haplotypes association analyses. Interestingly, common QTL regions were identified for all the analysed traits except for backfat thickness at 75 kg (BFT75).

Three QTL regions (M2, M5 and M13), on porcine chromosomes SSC1, SSC2 and SSC7, appeared segregating in all three backcrosses, which may indicate an Iberian origin, specifically for M2 and M13 regions, which have not been previously identified. The M2 region (SSC1:0.7-3Mb) affecting body weight has not been previously described, the closest QTL for body weight on this chromosome is around 10 Mb in Landrace-Duroc-Yorkshire dams [52]. Within this region, a functional candidate gene, *PDE10A* (*Phosphodiesterase 10A)*, was annotated according to the current porcine genome annotation version (*Sscrofa11*.*1*). The *PDE10A* encodes for a phosphodiesterase implicated in the regulation of energy homeostasis and it has been proposed as a promising candidate target for the treatment of obesity and diabetes [53]. One SNP within the gene was detected in the mentioned RNA-Seq analysis segregating in all three backcrosses, *rs321518792*, following Sscrofa10.2 annotation, however in the new Sscrofa11 is not mapped.

The M5 region in SSC2:3-9Mb affecting loin bone-in weight also appeared segregating in all three backcrosses, but this region has been previously identified in other animal material such as Large White, Meishan, Pietrain, Leicoma and Landrace [54–56]. Within the region, nine functional candidate genes were annotated according to the current porcine genome annotation version (*Sscrofa11*.*1*) and another one showed expression differences according to RNA-Seq data. Among those genes, the *ACTN3* (*Actinin alpha 3*) constitutes an interesting candidate to regulate muscle performance through the calcineurin signalling [57] and the *DHCR7* gene (*7-dehydrocholesterol reductase*) associated with obesity through vitamin D pathway [58,59]. Four SNPs in *DHCR7* gene segregating in the three backcrosses were detected in our RNA-Seq data, two of them are missense, rs334446395 and rs323256060, which could be interesting to further study in association analysis.

The M13 region in SSC7: 3Mb affecting shoulder weight and segregating in all three backcrosses has not been previously described, the closest QTL is around 20–40 Mb in Large White x Meishan [55]. Within this region, it is worth to highlight the *DSP* gene (*Desmoplakin*), which encodes the molecular component responsible of cell adhesion and signalling, including muscle cells, which may determine muscle properties [60]. Candidate SNPs within these genes have been identified from the RNA-Seq data as shown in Table 7, highlighting the missense one identified in *DSP*, no annotated previously in the SNP database but segregating in BC_PI.

Three other QTL regions (M4, M7 and M9) appeared segregating in BC_DU and BC_PI backcrosses, both backcrosses share the Torbiscal (red Iberian strain) parental male origin, different than the BC_LD, where Guadyerbas (black hairless strain) is the parental male origin. The M4 QTL has been widely reported in different pig populations including Duroc, Large White, Meishan, Bershire, etc… [54, 61, 62]. In the current study segregation of this QTL was not detected in the BC_LD backcross. Within the region fifteen functional candidate could be annotated, highlighting again the *DHCR7* gene. The M7 region affecting backfat thickness is the same or close to the well-known FAT1 QTL [63, 64], segregating in the BC_DU and BC_PI backcrosses in the current study, although different QTL regions for growth and fatness have been identified around the same SSC4 region [65, 31]. Several candidate genes underlying this QTL have been analysed, however the causal mutation has not been identified yet [66–69]. In the current analysis, two functional candidate genes were annotated, and another one showing expression differences according to our RNA-Seq data. Highlighting, the *CSF1* gene (*Colony stimulating factor 1*), which encodes a myokine, involved in lipid metabolism and cholesterol levels [70]. Two candidate SNPs segregating in BC_PI and BC_DU have been identified from the RNA-Seq data, one missense variant and one in 5’ UTR. The M9 region for ham weight appeared segregating in BC_DU and BC_PI, but close to the significance in the BC_LD (P-value = 0.086). These regions have been previously associated to ham weight in Pietrain and Large White [71]. Within the selected candidate genes, the *FTO* gene (*Alpha-ketoglutarate dependent dioxygenase*) has been associated with porcine carcass traits [72] and it is target of multiple obesity-related studies in human [73, 74], however candidate SNPs within these last two genes could not been identified from the RNA-Seq segregating in BC_DU. Another strong candidate is the *NEU3* gene (*Neuraminidase 3*), which encodes a marker of insulin sensitivity, regulated by fatty acid metabolism [75] and where one 3´UTR variant has been identified segregating in the BC-DU.

Two QTL for SW (M6 and M11) appeared segregating in the BC_LD and BC_DU backcrosses. The M6 region match with previous QTL for ham muscle weight described in our previous studies in the IBMAP population [65]. Within this region there is a strong candidate gene, *COL14A1* (*Collagen type XIV alpha 1 chain*), which encodes a collagen chain involved in the regulation of fibrillogenesis and showing expression differences in our RNA-data and in previous transcriptome analysis in pig [76]. Moreover, four SNPs within the gene have been identified from our RNA-Seq data, one of them, rs321008722, in the 3’UTR region, which could potentially alter transcript expression. The M11 region has been associated with shoulder weight in a Pietrain x Large White x Landrace x Leicoma experimental population [77]. The region contains the *MFN2* gene (*Mitofusin 2*), which encodes a mitochondrial membrane protein involved in the regulation of muscle cell proliferation, and it plays a role in the pathophysiology of obesity [78]. Five SNPs within this gene have been identified in our RNA-Seq data, at the 3’UTR and segregating in the tree backcrosses.

The common M14 region appeared segregating in the BC_LD and BC_PI, where a QTL for hind leg conformation has been previously mapped in Landrace [79]. Here, a powerful positional and functional candidate is located, the *CCNY* gene (*cyclin Y*), which encodes for a member of the cyclin family involved in the regulation of adipogenesis and lipid production [80]. Interesting candidate SNPs within this gene have also been identified from our RNA-Seq data, which may alter transcription regulation.

Additionally, regions only identified in one of the three backcrosses were also further examined due to the potential relevant information for specific pig breeds.

In the BC_LD background, four QTL regions (LD1, LD2, LD3 and LD4) were identified on SSC5, SSC6, SSC11 and SSC16, for premium cut weights or backfat thickness. Except LD1, which is not reported in the pig QTL database, the rest of the regions have been reported in previous studies involving Landrace pigs (LD2 in Li et al., [81]; LD3 in Fernandez et al. [31]; LD4 in Onteru et al. [82]). The current analyses showed that they are not segregating in the BC_DU and BC_PI, which together with previous results suggest Landrace specific origin. Within these regions we have identified interesting candidate genes such as *SALL1 (Spalt like transcription factor 1)*, key factor for muscle development [83] and *PANK3 (Pantothenate kinase 3)*, regulator of CoA biosynthesis, essential for lipid and energy metabolism [84]. Interestingly, variants near to *ssc-mir-103-1* miRNA considered *PANK3* regulators and recently associated with the analysed traits in pig [85] were identified and could be considered strong candidate variants to be analysed in further studies.

In the BC_DU background, four QTL regions (DU1, DU2, DU3 and DU4) were identified in the backcross specific GWAS, but another three regions (M8, M10 and M15) could be identified as BC-DU specific for LBW, BFTS and IMF during the haplotype association analysis performed to validate common QTL segregation. Several QTL for backfat thickness have been described matching DU1 region in different genetic backgrounds (Asian, European, commercial and traditional pig breeds) [86–88], however, in the current study we could identify segregation only in the BC_DU. In the same way, several QTL have been described matching DU3 region in different genetic backgrounds, around *UCP3* gene (*Uncoupling protein 3*) [86,89,90], also matching DU2 [91], M8 [92] and M10 [77]. However, no QTL has been previously identified for shoulder weight around DU4 region (SSC15:8-10Mb). Within these QTL regions there are strong functional candidates to carry the causal mutation, as *LMO4* gene (*LIM domain only 4*), which modules proliferation and differentiation of preadipocytes [93], *FTO* [94] and *NEU3* gene [75].

Finally, in the BC_PI background, four QTL regions (PI1, PI2, PI3 and PI4) were identified in the backcross specific GWAS, but another two regions, M1 and M3, were also validated as BC-PI specific for LBW during the haplotype association analysis performed to validate common QTL segregation. All these regions match with previously described QTL: PI1 in Lents et al. [95]; PI2 in Walling et al. [96]; Tortereau et al. [54]; PI3 in Cherel et al. [97]; P4 in Terenina et al. [98]; M3 in Malek et al. [99]; except for M1. As before, within these QTL regions there are strong functional candidates to carry the causal mutation such as the *PDR3B* gene (*Phosphodiesterase 3B*), which encodes one of the enzymes involved in the regulation of energy homeostasis, in adipocytes, hepatocytes, hypothalamic cells and β cells [100], however candidate SNPs within this gene has not been identified from our RNA-Seq data.

Moreover, in order to test the SNP number effect, an additional GWAS comparison within BC_DU using different SNPs number, employing 60K or 650K SNP chip genotyping information was conducted (S4 Table). The main results for the QTL scan resulted similar when using both, 60K and 600K platforms, obtaining the same significant QTL regions. The principal difference lies in the number of TAS within each region, being larger when employing the 650K chip, as expected, and leading to a slight difference for the QTL region boundaries.

The design and analysis methodology employed in the current study, using different genetic backgrounds and high-density SNP data, in SNP GWAS and haplotype association analyses in merged and specific datasets has allowed us to determine nine QTL regions for growth, premier cut weights and intramuscular fat validated in different genetic backgrounds supporting their reliability. Additionally, some backcross-specific QTL regions were also identified. Finally, the integration of RNA-Seq data and functional annotation has facilitated the selection of strong candidate genes such as *PDE10A*, *DHCR7*, *MFN2*, *FTO*, *CCNY* and *SALL1* and gene variants segregating that need to be further investigated in order to identify actual causal mutations.

## Supporting information

S1 Fig Graphical representation of the minimum allele frequency (MAF). SNPs included in each of the datasets analyzed (BC_LD, BC_DI, BC_DU and Merged dataset).
(TIF)

S1 Table Significant SNP associations (q-value <0.05) identified in the merged and backcross specific datasets for body weight at 150 days (BW150), backfat thickness at slaughter (BFTS), backfat thickness at 75 kg (BFT75), ham weight (HW), shoulder weight (SH), loin bone-in weight (LBW) and intramuscular fat (IMF): SNP ID, chromosome, position, reference allele, alternative allele, additive effect, standard error, *P-value* and trait. (XLSX)

S2 Table Haplotype description. Description for each common QTL region and segregation within the different datasets.
(XLSX)

S3 Table List of candidate SNPs. SNPs selected from those SNPs identified in parallel RNA-Seq studies within positional and functional candidate genes.
(XLSX)

S4 Table Results of the GWAS. Analysis carried out with the 650K genotyping chip data for the regions identified with the 60K porcine chip in the BC_DU backcross.
(XLSX)
