# Supplementary figures and images for: Using genome wide association studies to identify common QTL regions in three different genetic backgrounds based on Iberian pig breed
Source: PLoS One. 2018 Mar 9;13(3):e0190184. doi: 10.1371/journal.pone.0190184 (PMC5844516; doi:10.1371/journal.pone.0190184)

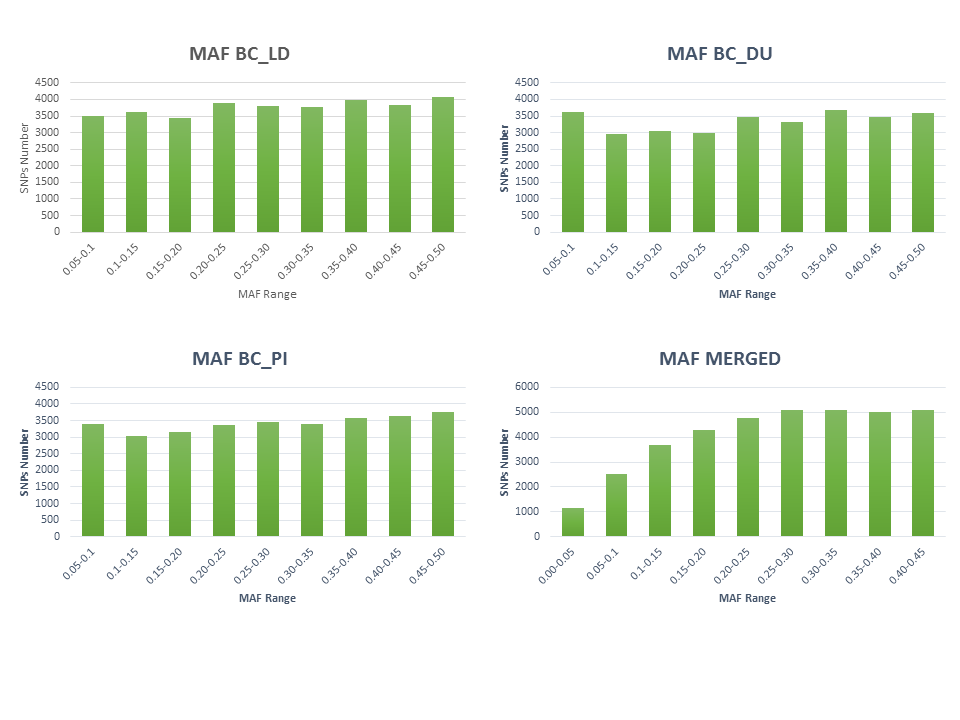

Supplement: S1 Fig — SNPs included in each of the datasets analyzed (BC_LD, BC_DI, BC_DU and Merged dataset). (TIF) [file pone.0190184.s001.tif]
